# Assembly and comparative analysis of the complete mitochondrial genomes of five *Thaxterogaster* species (*Cortinariaceae*, *Basidiomycota*): Insights into mitochondrial genome evolution and intrageneric phylogenetic relationships

**DOI:** 10.3897/mycokeys.140.205520

**Published:** 2026-09-18

**Authors:** Li-Ye Ou, Meng-Le Xie, Yi Li, Chang-Tian Li

**Affiliations:** 1 Engineering Research Center of Chinese Ministry of Education for Edible and Medicinal Fungi, Jilin Agricultural University, Changchun 130118, Jilin, China Engineering Research Center of Chinese Ministry of Education for Edible and Medicinal Fungi, Jilin Agricultural University Changchun China; 2 College of Mycology, Jilin Agricultural University, Changchun 130118, Jilin, China College of Mycology, Jilin Agricultural University Changchun China; 3 Key Laboratory of Edible Fungal Resources and Utilization (North), Ministry of Agriculture and Rural Affairs, Jilin Agricultural University, Changchun 130118, Jilin, China Key Laboratory of Edible Fungal Resources and Utilization (North), Ministry of Agriculture and Rural Affairs, Jilin Agricultural University Changchun China; 4 School of Food Science and Engineering, Yangzhou University, Yangzhou 225127, Jiangsu, China School of Food Science and Engineering, Yangzhou University Yangzhou China; 5 Innovation Center for Edible Fungi Resources and Application Technology, Xizang Autonomous Region, Lhasa 850000, Xizang, China Innovation Center for Edible Fungi Resources and Application Technology Lhasa China

**Keywords:** *
Agaricales
*, comparative genomics, mitochondrial genetics, mitogenomic evolution, phylogenomics

## Abstract

The generic circumscription and taxonomic rank of *Thaxterogaster* have undergone substantial revisions over time, yet the intrageneric phylogenetic affinities of this genus remain contentious. While mitochondrial genomes are effective molecular markers for phylogenetic analyses, systematic studies based on complete mitogenomes of this genus are still lacking. In this study, we assembled and annotated the complete mitogenomes of five *Thaxterogaster* species and performed comparative genomic and phylogenetic analyses. The five mitogenomes are all circular molecules, ranging from 35,902 to 106,732 bp in length, with GC contents of 24.35%–29.15%. All mitogenomes shared a conserved repertoire of 15 core protein-coding genes, two rRNA genes, and 25–26 tRNA genes. The core gene complement and gene order are relatively conserved among the five species, whilst genome size, intron load, repeat sequence composition, and local syntenic architecture vary markedly. Phylogenetic analyses based on the mitochondrial core genes of 51 species revealed that the five *Thaxterogaster* species formed a well-supported distinct clade within *Cortinariaceae*, closely related to the clade comprising *Hymenogastraceae* and *Strophariaceae*. Collectively, our findings enhance the understanding of mitogenomic structural and evolutionary features in *Thaxterogaster* and provide a useful reference for future studies into mitogenome evolution in *Cortinariaceae* and allied taxa.

## Introduction

The mitochondrial genome is an important molecular marker for phylogenetic studies of eukaryotes and has been extensively utilized to clarify phylogenetic relationships and species evolution of animals. For example, the phylogenetic relationships and divergence history of major *Mantodea* lineages remain uncertain; mitochondrial phylogenomic data have been used to construct a phylogenetic framework and estimate divergence times, providing molecular evidence for understanding the evolutionary history of major mantodean lineages (Ma et al. 2023). Similarly, the phylogenetic relationships within *Rhiniinae* have long remained poorly resolved. A recent analysis based on complete mitogenomes of representative species robustly supported the monophyly of this subfamily and clarified its internal relationships (Li et al. 2026). Phylogenies reconstructed based on mitochondrial genes often show incongruence with results from nuclear and chloroplast genes, which restricts the application of mitogenomes in plant phylogenetic and evolutionary research. After eliminating the interfering factors in mitochondrial coding sequences, Hu et al. (2025) estimated that the crown group of land plants originated in the Neoproterozoic and angiosperms arose in the Triassic; this temporal framework is consistent with nuclear genomic studies.

Fungal mitogenomes usually contain a relatively conserved set of core protein-coding genes (PCGs), which provide useful molecular information for phylogenetic analyses of different fungal groups (Kulik et al. 2021). At the same time, fungal mitogenomes show substantial variation in genome size, gene arrangement, intron load, open reading frames (ORFs), repeat sequences and local synteny (Aguileta et al. 2014; Kouvelis et al. 2023). Studies on comparative fungal mitogenomics have shown that mitogenome data can be used to analyse gene rearrangements, intron dynamics and phylogenetic relationships, and can provide molecular evidence for phylogenetic analyses of basidiomycete fungi (Li et al. 2020; Ma et al. 2025).

*Thaxterogaster* Singer, belonging to *Cortinariaceae (Basidiomycota)*, represents an important group of ectomycorrhizal fungi (Liimatainen et al. 2022). Species of this genus form symbiotic associations with the roots of host plants and play important roles in the maintenance of forest ecosystems. Additionally, several *Thaxterogaster* species possess edible properties and potential medicinal value (Xie et al. 2023). The taxonomic status of *Thaxterogaster* has long been debated. To date, phylogenetic studies of this genus mainly rely on multigene datasets, such as nrITS, nrLSU, RPB1 and RPB2 (Soop et al. 2025; Wang et al. 2025). Previous studies combining nuclear genomic and multilocus sequence data have re-established *Thaxterogaster* as an independent genus (Liimatainen et al. 2022). However, other studies have expressed different views on the splitting of *Cortinarius* s.l., indicating that the systematic framework of this group still requires additional independent molecular evidence (Gallone et al. 2024). As a genetic system independent of the nuclear genome, the mitogenome can provide complementary phylogenetic evidence for *Thaxterogaster* and other allied taxa within *Cortinariaceae*. However, systematic comparative analyses of mitogenomic evolution across this group remain sparse.

In this study, five *Thaxterogaster* species, namely *T. borealicremeolina*, *T. crassimultiformis*, *T. lavendulaceus*, *T. sinopurpurascens* and *T. sinoscaurus*, were selected, and their complete mitogenomes were assembled and annotated. This study aimed to: (1) systematically characterize the basic mitogenomic features of five *Thaxterogaster* species; (2) comprehensively compare structural variations in genome size, gene composition, introns, repeat sequences and local synteny; and (3) evaluate the applicability of mitochondrial core PCGs for resolving the phylogenetic relationships within *Thaxterogaster* and other taxa in *Agaricales*.

## Materials and methods

### Sample sources, DNA extraction and sequencing

The samples in this study represented five species of *Thaxterogaster*. All samples were taxonomically identified based on nrITS sequence comparisons with type specimen. Total DNA extraction and whole-genome sequencing were performed using either pure strains or dried specimens. Voucher specimens were deposited in the Herbarium of Mycology of Jilin Agricultural University (HMJAU), and the corresponding strains were preserved at the Jilin Agricultural University Edible Mushroom Germplasm Resource Conservation and Utilisation Center.

Genomic DNA was extracted from *Thaxterogaster* samples using a genomic DNA extraction kit (CWBIO, Beijing, China) per the manufacturer’s instructions. DNA purity (OD_260_/_280_) and concentration (ng/μL) were measured using a NanoDrop spectrophotometer and a Qubit fluorometer, respectively. Agarose gel electrophoresis was performed to further verify DNA integrity. After quality assessment, all qualified samples were sent to Shanghai Lingen Biotechnology Co., Ltd. for whole-genome sequencing.

Sequencing libraries for the five species were constructed using the Illumina TruSeq™ Nano DNA Sample Prep Kit and subjected to paired-end sequencing on the Illumina HiSeq 4000 platform. In addition to Illumina short reads, we generated PacBio continuous long reads (CLRs) using the PacBio RS II platform for *T. borealicremeolina*; this long-read dataset aided *de novo* assembly and improved overall genome completeness.

### Mitogenome assembly and validation

All raw sequencing datasets were preprocessed for quality control and filtering prior to mitogenome assembly. Raw Illumina paired-end reads were processed and filtered using fastp v0.23.2 (Chen et al. 2018). The filtered Illumina data were used for mitogenome assembly and validation. PacBio CLR data were used directly for subsequent genome assembly after quality assessment.

For *T. borealicremeolina*, de novo assembly was initially performed using Flye v2.9.6-b1802 based on the PacBio CLR data (Kolmogorov et al. 2019). Candidate mitochondrial contigs were screened based on sequencing depth, sequence length, and evidence of circularisation. The corresponding Illumina paired-end reads were then mapped back to the candidate sequence using BWA v0.7.19 (Li and Durbin 2009). The alignment results were subsequently sorted with SAMtools v1.13 (Li et al. 2009) and polished using Pilon v1.24 to improve assembly accuracy by correcting residual base-level errors and small insertions/deletions (indels) (Walker et al. 2014).

For the remaining four species, *T. crassimultiformis*, *T. lavendulaceus*, *T. sinopurpurascens* and *T. sinoscaurus*, mitogenome assembly was performed from Illumina paired-end reads using GetOrganelle v1.7.7.1, with the mitogenome sequence of *T. borealicremeolina* as the reference (Jin et al. 2020). The resulting assembly graphs were visualised in Bandage v0.8.1 to confirm the circular structure of the assembled mitogenomes (Wick et al. 2015). The complete mitogenomes of the five *Thaxterogaster* species analysed in this study have been deposited in GenBank under accession numbers PZ409756–PZ409760.

### Mitogenome annotation and visualisation

The assembled mitogenome sequences were annotated using MITOS v2.1.9 and MFannot web server, followed by manual curation (Bernt et al. 2013; Lang et al. 2023). tRNA genes were predicted with tRNAscan-SE 2.0 web server (Chan and Lowe 2019). Circular mitogenome maps were ultimately generated using the OGDRAW web server (Greiner et al. 2019).

### Validation of mitochondrial genome assemblies and annotations

Illumina clean reads derived from each sample were remapped to their respective final mitochondrial genome assemblies using BWA v0.7.19 (Li and Durbin 2009). Primary alignments were subsequently processed with SAMtools v1.13 (Li et al. 2009) to generate mapping statistics and calculate genome-wide coverage and sequencing depth. Genome circularity was assessed using reads spanning the terminal junction. PacBio CLR reads were used for *T. borealicremeolina*, whereas Illumina reads were employed for the remaining four species.

Annotation validation was performed on the basis of GenBank output files. The presence and uniqueness of the 15 conserved mitochondrial PCGs were examined, and CDS reading-frame integrity, internal stop codons and translation consistency were assessed using the fungal mitochondrial genetic code (NCBI translation table 4). The rRNA genes *rns* and *rnl* were initially annotated in MITOS, then manually validated against well-annotated homologous sequences from *Hebeloma cinnamomeum* (PZ270485.1) and *Gymnopilus junonius* (NC_057300.1) using MAFFT v7.525 (Katoh and Standley 2013) and BLASTN v2.15.0+ (Camacho et al. 2009).

### Comparative mitogenomic analysis

Comparative analyses were performed on the core protein-coding genes (PCGs) in the mitogenomes of the five *Thaxterogaster* species. Gene length, start codon, stop codon and GC content were calculated for each gene. Nucleotide composition skews of PCGs were computed according to the following formulas AT-skew = (A – T) / (A + T) and GC-skew = (G – C) / (G + C). After removal of terminal stop codons from all coding sequences (CDSs), relative synonymous codon usage (RSCU) values were calculated using CodonW v1.4.2, with the fungal mitochondrial genetic code NCBI translation table 4 applied for analyses (Peden 1999).

To compare genetic distances and substitution rate variation among the 15 core PCGs, each gene sequence was first translated into amino acid sequences based on fungal mitochondrial genetic code and aligned using MUSCLE v5.3 (Edgar 2022). The resulting amino acid alignments were back-translated to generate codon-based nucleotide alignments. Using these codon alignments, Ka, Ks and Ka/Ks values were estimated with KaKs_Calculator v3.0 under the YN model (Yang and Nielsen 2000; Zhang 2022), and computed pairwise genetic distances using the Kimura 2-parameter (K2P) model (Kimura 1980). All resulting data were statistically summarised and visualised using Python scripts.

### Intron classification and analysis

Pearson correlation analysis was employed to evaluate the association between mitogenome size and intron number. Introns within the *cox1* gene of 12 *Agaricales* mitogenomes were classified into positional classes (Pcls) following the protocol proposed by Férandon et al. (2010). The *cox1* sequence of *T. borealicremeolina* was used as the reference, and the amino acid sequences encoded by *cox1* of all examined species were aligned using MAFFT v7.525 (Katoh and Standley 2013). Intron insertion sites were subsequently mapped to homologous positions of the reference sequence. Introns inserted at the same position were assigned to the same Pcl and regarded as homologous introns.

### Repeat sequence analysis

Simple sequence repeats (SSRs), tandem repeats and interspersed repeats were detected across all mitogenomes using the MISA web server, Tandem Repeats Finder (TRF) and UGENE v53.1, respectively (Benson 1999; Okonechnikov et al. 2012; Beier et al. 2017). The resulting repeat datasets were processed and visualised with Python scripts to characterise differences in repeat composition and abundance among the five species.

### Prediction and statistical analysis of putative RNA editing sites

Putative C-to-U RNA editing sites within the 15 core PCGs of the five *Thaxterogaster* species were predicted using Deepred-mt, a tool based on convolutional neural networks (Edera et al. 2021). Only high-confidence sites with probability values above 0.9 were retained for further analyses. Editing-site density was normalised by the number of cytosines in each gene, and deviations from a cytosine-proportional distribution were assessed using a chi-square goodness-of-fit test (Pearson 1900), with gene-specific deviations evaluated using two-sided binomial tests followed by Holm–Bonferroni correction (Holm 1979). The functional effects of predicted sites were compared with those of the remaining non-predicted cytosines using Fisher’s exact test (Fisher 1922). Cross-species conservation at homologous amino acid positions was assessed using 10,000 permutations that preserved the species- and gene-specific numbers of predicted sites, with empirical P values calculated using a plus-one correction (Phipson and Smyth 2010). Because the predictions were not validated using transcriptomic data, all identified sites were treated as putative.

### Phylogenetic analysis

Publicly available fungal mitogenome sequences were retrieved from NCBI and combined with the five newly generated *Thaxterogaster* mitogenomes to construct the final phylogenetic dataset. Detailed information for all 51 sampled taxa is provided in Suppl. material 1: table S1. Based on individual mitogenome annotations, amino acid sequences of the 15 shared core PCGs were extracted and aligned using MUSCLE v5.3 (Edgar 2022). The resulting alignments were trimmed with trimAl v1.2rev57 (Capella-Gutiérrez et al. 2009) and subsequently concatenated for downstream phylogenetic analyses. Sequence concatenation and partition file generation were performed using PhyloSuite v2 (Zhao et al. 2025).

For phylogenetic reconstruction, the optimal partitioning scheme and amino acid substitution models were evaluated using the ModelFinder module implemented in IQ-TREE v3.0.1, with the partition merging strategy MFP+MERGE under the Bayesian Information Criterion (BIC) (Kalyaanamoorthy et al. 2017; Wong et al. 2026). A maximum likelihood (ML) tree was reconstructed based on the best-fit models (Wong et al. 2026). Bayesian inference (BI) analysis was performed using MrBayes v3.2.7a, with the dataset partitioned by the 15 core PCGs; each partition was assigned a mixed amino acid model prior, and among-site rate variation was modeled using a Gamma distribution (Ronquist et al. 2012). Two independent Markov chain Monte Carlo (MCMC) runs, each comprising four chains, were conducted for approximately 1.6 million generations, with trees sampled every 1,000 generations. The first 25% of the sampled trees were discarded as burn-in. Convergence was assessed using the average standard deviation of split frequencies, which reached 0.003221. The resulting trees were visualised and annotated using the iTOL web server (Letunic and Bork 2024).

### Mitogenome collinearity analysis

BLASTN v2.15.0+ was used to perform homology searches among the complete mitogenomes of the 12 related species (Camacho et al. 2009). The resulting homologous regions were filtered and formatted as input files for MCScanX v1.0.0, which was then used for multiple synteny analysis and visualization (Wang et al. 2012). In addition, Mauve v2.4.0 was employed to compare syntenic relationships and detect genomic rearrangements among the five *Thaxterogaster* mitogenomes (Darling et al. 2004). The Mauve alignment results were subsequently visualised using a custom Python script.

## Results

### General features of *Thaxterogaster* mitogenomes

**Figure 1.**
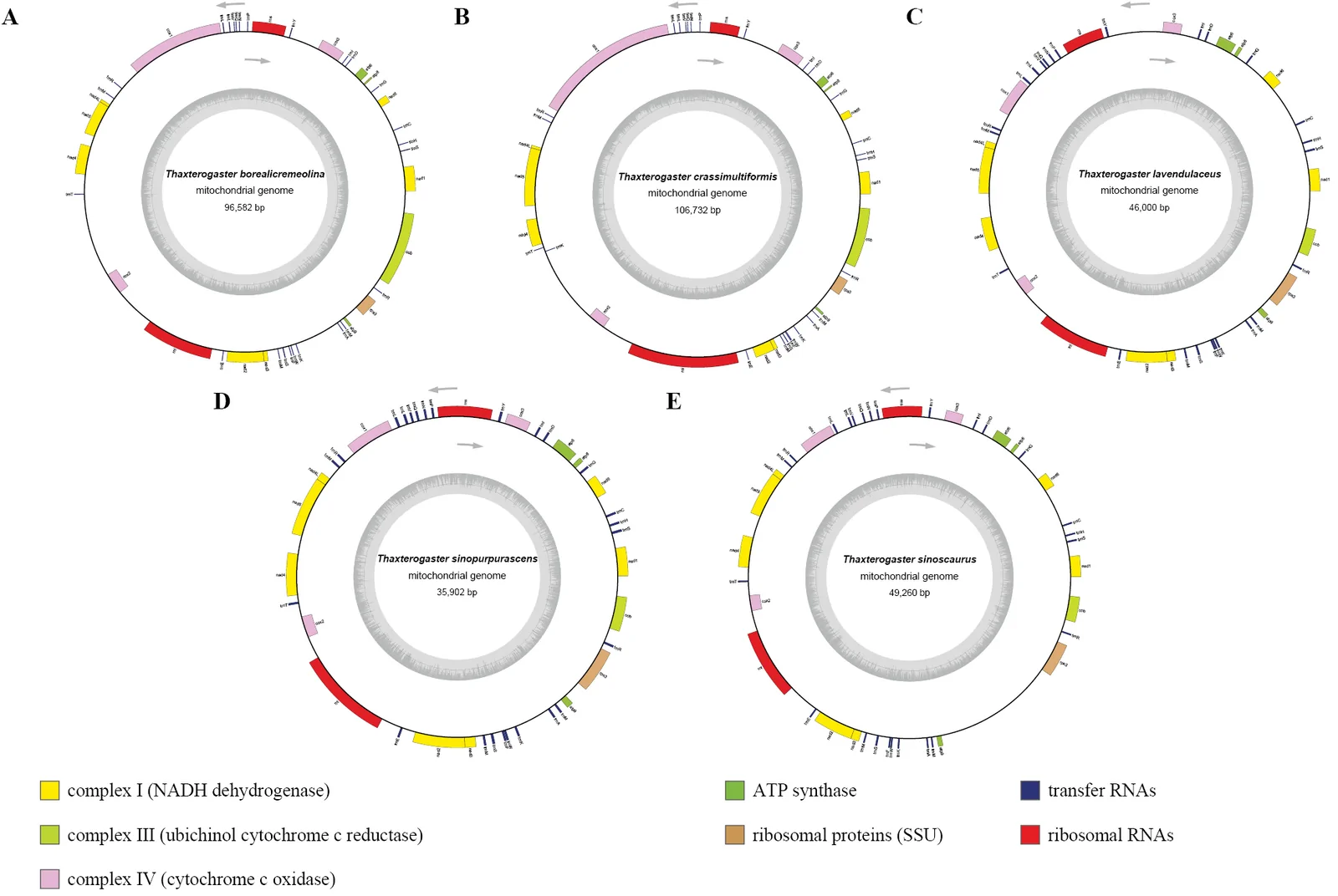
Circular maps of five *Thaxterogaster* mitogenomes. **A**. *T. borealicremeolina*; **B**. *T. crassimultiformis*; **C**. *T. lavendulaceus*; **D**. *T. sinopurpurascens*; **E**. *T. sinoscaurus*. All five mitogenomes contain 15 core PCGs, two rRNA genes and 25–26 tRNA genes. The inner ring denotes GC content. Genes encoded on the reverse and forward strands are shown on the inner and outer sides of the circular map, respectively.

In this study, the complete mitogenomes of five *Thaxterogaster* species were newly assembled and annotated: *T. borealicremeolina*, *T. crassimultiformis*, *T. lavendulaceus*, *T. sinopurpurascens* and *T. sinoscaurus* (Fig. 1). All five mitogenomes were complete circular molecules, with total lengths of 96,582, 106,732, 46,000, 35,902, and 49,260 bp, respectively. Of these species, *T. crassimultiformis* exhibited the largest mitogenome, whilst *T. sinopurpurascens* possessed the smallest. The GC contents of the five mitogenomes were 27.56%, 29.15%, 27.37%, 24.35% and 27.05%, respectively, with corresponding AT contents being consistently higher in all five species.

Each of the five mitogenomes contained 15 core PCGs, two rRNA genes and 25–26 tRNA genes (Fig. 1; Suppl. material 1: table S2). Specifically, the 15 core PCGs were detected in all five species, including *atp6*, *atp8*, *atp9*, *cob*, *cox1*, *cox2*, *cox3*, *nad1*, *nad2*, *nad3*, *nad4*, *nad4L*, *nad5*, *nad6* and *rps3*. The two rRNA genes identified were the *rns* and *rnl*.

Circular maps of the five mitogenomes illustrated the physical distribution of PCGs, rRNA genes and tRNA genes. Overall, all five species shared the same core PCG complement but differed in genome size and tRNA gene number (Suppl. material 1: table S2).

### Features of RNA genes

Two rRNA genes, *rns* and *rnl*, were identified in each of the five *Thaxterogaster* mitogenomes (Suppl. material 1: table S2), with mean lengths of 2,302 and 5,455 bp, respectively. A total of 25–26 tRNA genes were annotated across all five species, covering the full set of 20 standard amino acids, with additional isoaccepting copies present in some species. *Thaxterogaster crassimultiformis* possessed 26 tRNA genes, whilst the other four species each contained 25 tRNA genes. Using *T. crassimultiformis* as a representative example, all 26 tRNA sequences were predicted to adopt canonical cloverleaf secondary structures (Suppl. material 2: fig. S1), with lengths ranging from 71 to 86 bp. Across all five species, tRNA gene lengths ranged from 71 to 87 bp, with *trnS* (TGA) being the longest, likely due to its extended variable arm. The positions of shared tRNA genes differed among the five mitogenomes. Non-canonical base pairing during tRNA secondary structure folding generated a total of 247 mismatched base pairs across five species: 48 in *T. borealicremeolina*, 46 in *T. crassimultiformis*, 49 in *T. lavendulaceus*, 53 in *T. sinopurpurascens*, and 51 in *T. sinoscaurus*. All mismatches were G–U wobble pairings, scattered throughout the stem domains of distinct tRNA transcripts. In the tRNA alignment, red indicates variable sites among the shared tRNA genes, whilst blue marks the additional *trnK* copy detected only in *T. crassimultiformis* (Suppl. material 2: fig. S1).

### Codon usage bias of core protein-coding genes

**Figure 2.**
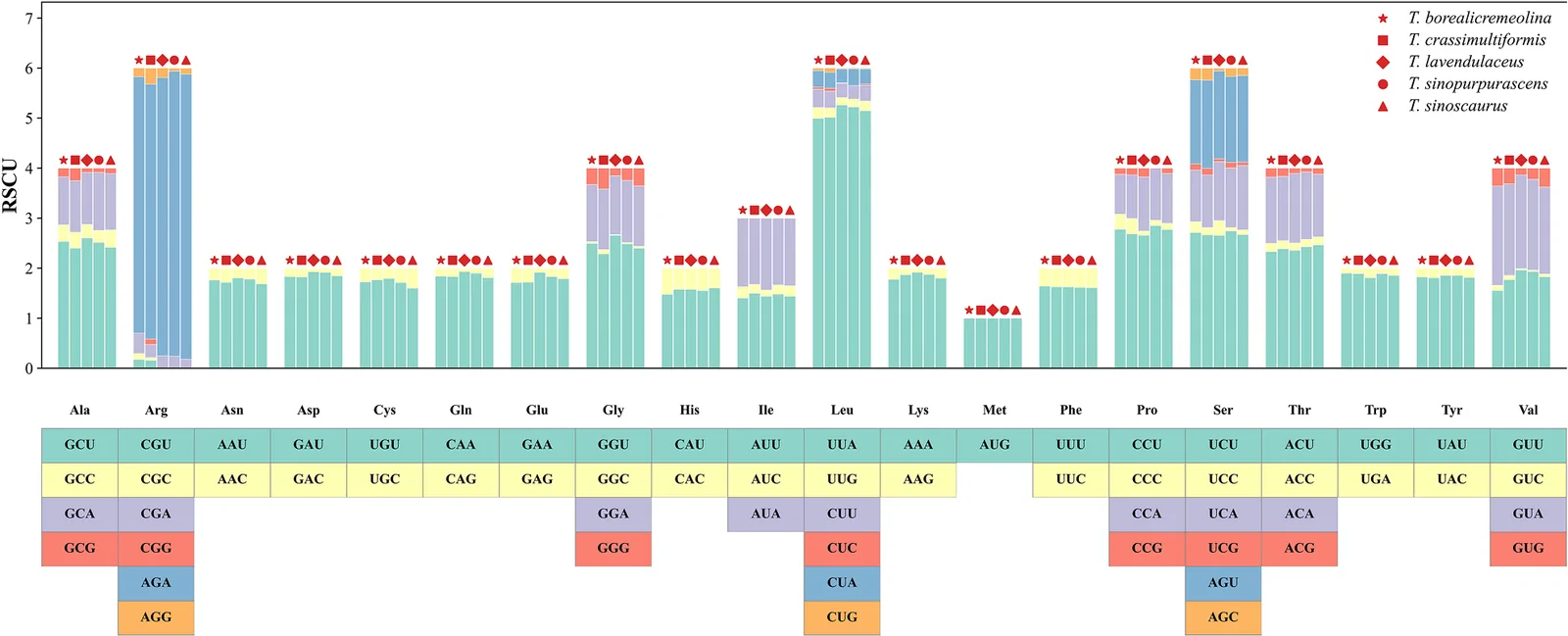
Codon usage patterns of mitochondrial core PCGs. Relative synonymous codon usage (RSCU) values of the 15 core PCGs are compared among the five *Thaxterogaster* species. The x-axis represents codon families; the boxes below indicate all codons encoding each amino acid.

The relative synonymous codon usage (RSCU) values of the 15 core PCGs were generally similar across the five *Thaxterogaster* species, with only minor interspecific differences (Fig. 2). After excluding terminal stop codons, 62 sense codons were included in the analysis, and the most frequently used codon for each amino acid is shown in Fig. 2. An RSCU value of 1 indicates no obvious codon usage bias, whilst values greater than 1 indicate relatively frequent usage of the corresponding codon.

The number of codons with RSCU values > 1 varied slightly among species: 25 in *T. borealicremeolina*, 26 in *T. crassimultiformis*, 27 in *T. lavendulaceus*, 27 in *T. sinopurpurascens*, and 26 in *T. sinoscaurus*. Across all species, the arginine codon AGA showed the highest RSCU values, ranging from 5.10 to 5.70, indicating a strong and conserved usage bias. The leucine codon UUA was the second most preferred (RSCU = 5.00–5.26), followed by CCU, UCU, GCU, GGU, and ACU. In contrast, methionine, encoded exclusively by AUG, showed an RSCU value of 1 across all five species. Overall, the five species displayed highly similar codon usage patterns, with a clear preference for A- or U-ending codons (Suppl. material 1: table S3).

### Variation, genetic distance and evolutionary rates of core protein-coding genes

The combined lengths of the 15 mitochondrial core PCGs ranged from 14,487 to 14,826 bp among the five *Thaxterogaster* species, and most homologous genes showed limited interspecific variation in length and nucleotide composition (Suppl. material 2: fig. S2). Although relatively greater length variation was observed in *nad2*, *rps3*, *nad4*, and *nad1*, the overall core PCG complement remained highly conserved. This suggests that the pronounced differences in total mitogenome size were not primarily attributable to variation in the core coding regions.

**Figure 3.**
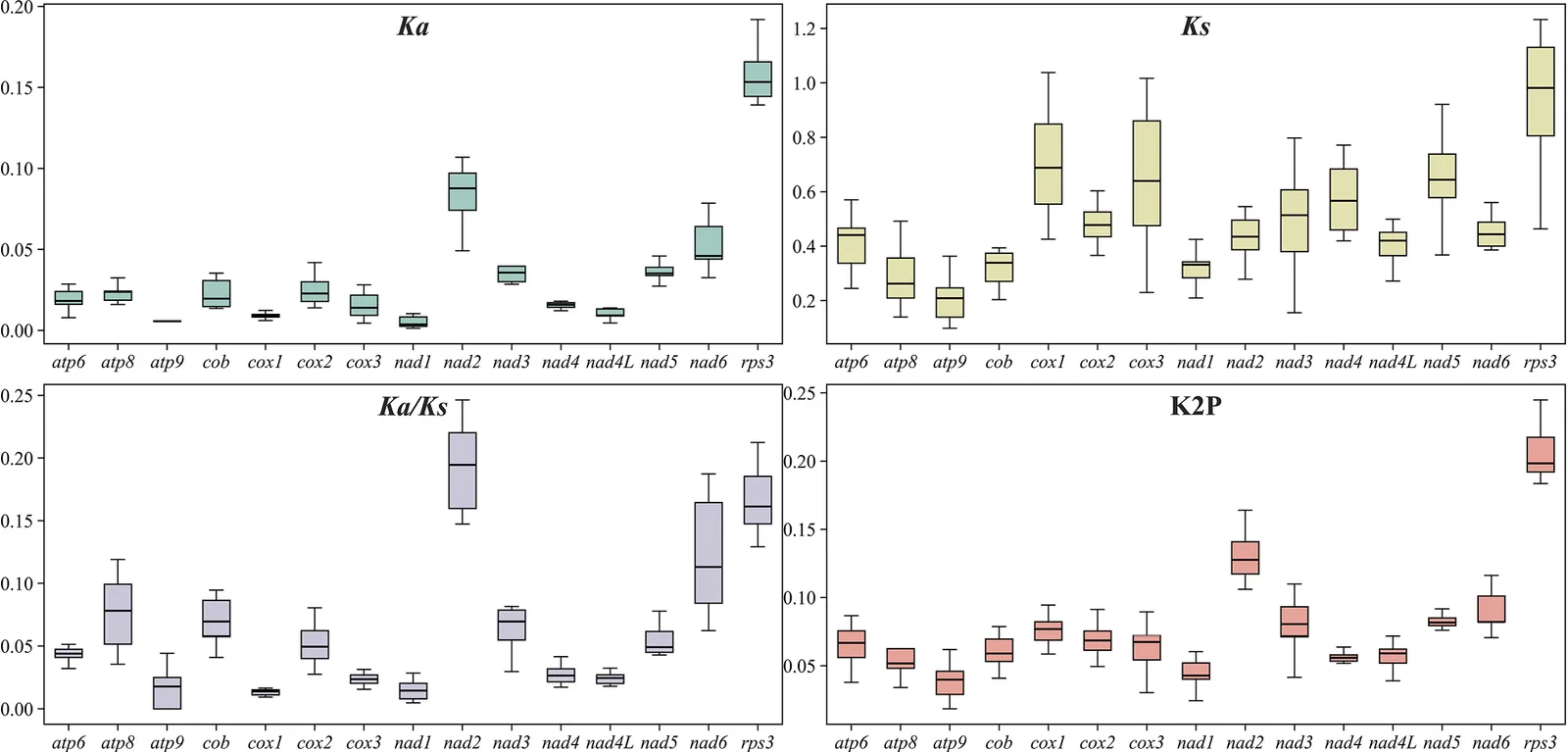
Genetic distances and substitution rates of mitochondrial core PCGs. Ka, nonsynonymous substitution rate; Ks, synonymous substitution rate; Ka/Ks, ratio of nonsynonymous to synonymous substitution rates; K2P, Kimura 2-parameter genetic distance.

Using the 15 core PCGs shared across all five *Thaxterogaster* mitogenomes, we calculated the nonsynonymous substitution rate (Ka), synonymous substitution rate (Ks), Ka/Ks ratio, and Kimura 2-parameter (K2P) pairwise genetic distance for each gene (Fig. 3). Among the core PCGs, *rps3* showed the highest mean Ka and Ks of 0.1546 and 1.0400, respectively, whilst *nad1* recorded the lowest mean Ka (0.0050) and *atp9* the lowest mean Ks (0.2078). The Ka/Ks ratios of all core PCGs were below 1.0, with *nad2* returning the highest average ratio of 0.1920, followed by *rps3* and *nad6* with ratios of 0.1609 and 0.1206, respectively.

K2P genetic distance analysis revealed that *rps3* possessed the greatest mean K2P value (0.2024), followed by *nad2* (0.1272) and *nad6* (0.0894), indicating elevated sequence divergence of these three genes across the five species. In contrast, *atp9* and *nad1* showed the lowest mean K2P values of 0.0390 and 0.0440, respectively, reflecting strong sequence conservation. Collectively, both Ka/Ks ratios and K2P distances consistently identified *rps3*, *nad2*, and *nad6* as the most variable genes, whilst *atp9* and *nad1* displayed minimal sequence divergence.

### Intron dynamics in mitogenomes

**Figure 4.**
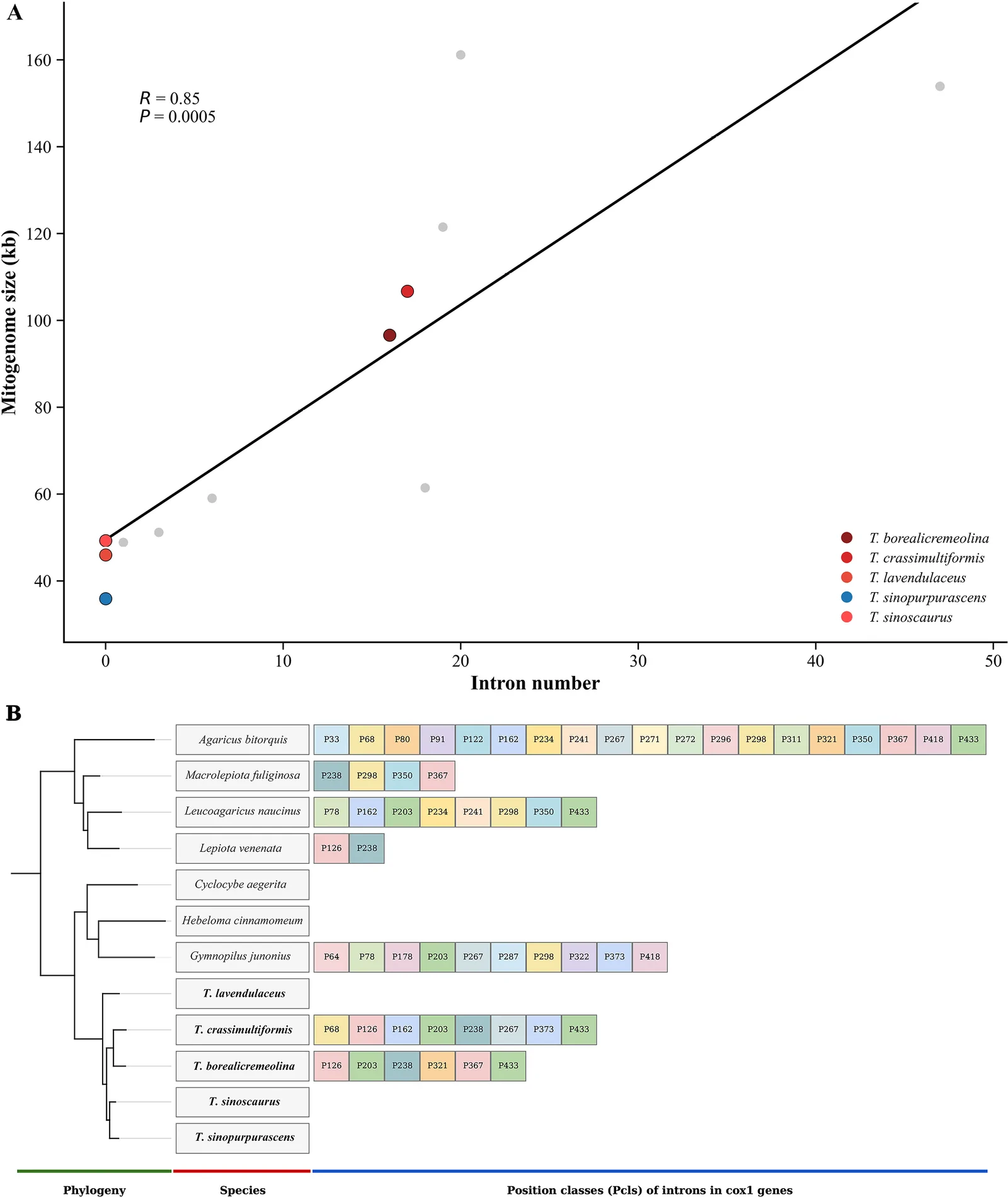
Intron dynamics in *Thaxterogaster* and related mitogenomes. **A**. Pearson correlation analysis between mitogenome size and total intron number; **B**. Distribution of Pcl types classified based on homologous insertion positions within the *cox1* gene. Coloured boxes indicate the presence of introns at the corresponding homologous *cox1* positions in each species. Species names and corresponding NCBI accession numbers are provided in Suppl. material 1: table S1.

Introns were unevenly distributed across distinct mitochondrial genes, with a striking bias toward *cox1*. Specifically, a total of 147 introns were annotated across the 12 mitogenomes examined here, 57 of which resided within the *cox1* locus and constituted 38.78% of all introns identified. Among all host genes, *cox1* accommodated the greatest intron count, followed by *cob* and *nad5*. Pearson correlation analysis revealed a significant positive correlation between total intron abundance and mitogenome size (R = 0.8465, P = 0.0005; Fig. 4A).

Using the *cox1* sequence of *T. borealicremeolina* as the reference, the 57 introns from the 12 mitogenomes were classified into 28 Pcl subtypes based on their insertion sites (Fig. 4B). Four subtypes, P203, P238, P298, and P433, were present across four taxa each, whilst a further 12 subtypes were unique to individual species.

Within the five *Thaxterogaster* species, eight Pcl subtypes were recovered in the *cox1* gene of *T. crassimultiformis*: P68, P126, P162, P203, P238, P267, P373, and P433. Six subtypes were detected in *T. borealicremeolina*: P126, P203, P238, P321, P367, and P433. By contrast, no introns were recovered from the *cox1* genes of *T. lavendulaceus*, *T. sinopurpurascens*, or *T. sinoscaurus*. *Thaxterogaster crassimultiformis* and *T. borealicremeolina* shared P126, P203, P238 and P433, whilst each species also harboured species-specific Pcl variants.

### Repeat sequence characteristics of mitogenomes

A survey of repeat sequences across the five mitogenomes identified a total of 192 simple sequence repeats (SSRs). Among the five species, *T. borealicremeolina* possessed the highest SSR count (87), whilst *T. sinopurpurascens* harboured the lowest (9). Mononucleotide repeats dominated the SSR repertoire in all species (Suppl. material 1: tables S4, S5). In addition, 94 tandem repeats were recovered, with repeat unit sizes ranging from 1 to 54 bp. The highest abundance of tandem repeats was recorded in *T. borealicremeolina* (33), and the lowest in *T. sinopurpurascens* (6) (Suppl. material 1: table S6). We also annotated 184 interspersed repeat pairs with a minimum length of 30 bp, comprising 107 forward repeats and 77 reverse-complement repeats. Among the five species, *T. crassimultiformis* exhibited the largest number of interspersed repeats (77 pairs), whilst *T. sinopurpurascens* retained the fewest (5 pairs) (Suppl. material 1: table S7). The overall distribution of all repeat sequences across the five mitogenomes is visualised in Suppl. material 2: fig. S3.

### Characteristics of putative RNA editing sites

Using predictions derived from the 15 PCGs, we recovered 318 high-confidence candidate C-to-U RNA editing sites in total (Suppl. material 1: table S8). These sites were distributed across 14 PCGs, with substantial variation in abundance among genes (Suppl. material 2: fig. S5A). *Cob* harboured the greatest count of predicted editing sites (56), followed by *nad5* (50), *nad2* (48), and *cox1* (33). No high-confidence sites were annotated within *nad4L*. After normalisation by cytosine content, the gene-wise distribution of predicted sites differed significantly from the cytosine-proportional expectation (χ^2^ = 69.35, df = 14, P = 0.000000002526; Suppl. material 1: table S10). Gene-specific two-sided binomial tests showed that *cob* was significantly enriched in predicted sites, whereas *cox3* was significantly depleted after Holm–Bonferroni correction. Predictions further indicated that several editing events could introduce premature stop codons in *nad1*, *nad2*, *nad3*, *nad4*, and *rps3*.

Of the 318 candidate sites, 287 were predicted to result in nonsynonymous changes: 276 resulted in amino acid substitutions, and 11 introduced stop codons. The remaining 31 sites caused synonymous substitutions (Suppl. material 2: fig. S5B). Amino-acid-changing substitutions accounted for 90.25% of the predicted sites, significantly exceeding the corresponding proportion of 82.29% among the remaining non-predicted cytosines (Fisher’s exact test, odds ratio = 1.99, P = 0.000115518; Suppl. material 1: table S10). Among the inferred amino acid changes, serine (Ser) to phenylalanine (Phe) was the most prevalent modification, followed by proline (Pro) to leucine (Leu) and serine (Ser) to leucine (Leu). Collectively, these candidate editing sites encompassed 26 distinct codon shifts, corresponding to 16 unique amino acid changes. Shifts converting hydrophilic residues to hydrophobic residues occurred most frequently, accounting for 57.86% of all events. Modifications retaining hydrophobic character constituted 32.08%, whilst hydrophilic-to-hydrophilic shifts, hydrophobic-to-hydrophilic shifts and stop-codon-generating alterations made up 3.46%, 3.14% and 3.46%, respectively (Suppl. material 1: table S9).

Cross-species mapping identified 138 homologous site groups, of which 77 were shared by at least two species. This number was significantly greater than the permutation expectation of 21.20 (95% permutation interval = 14–29; empirical P < 0.0001; Suppl. material 1: table S10), indicating greater conservation of homologous predicted sites than expected by chance.

### Mitogenome synteny and gene rearrangement features

**Figure 5.**
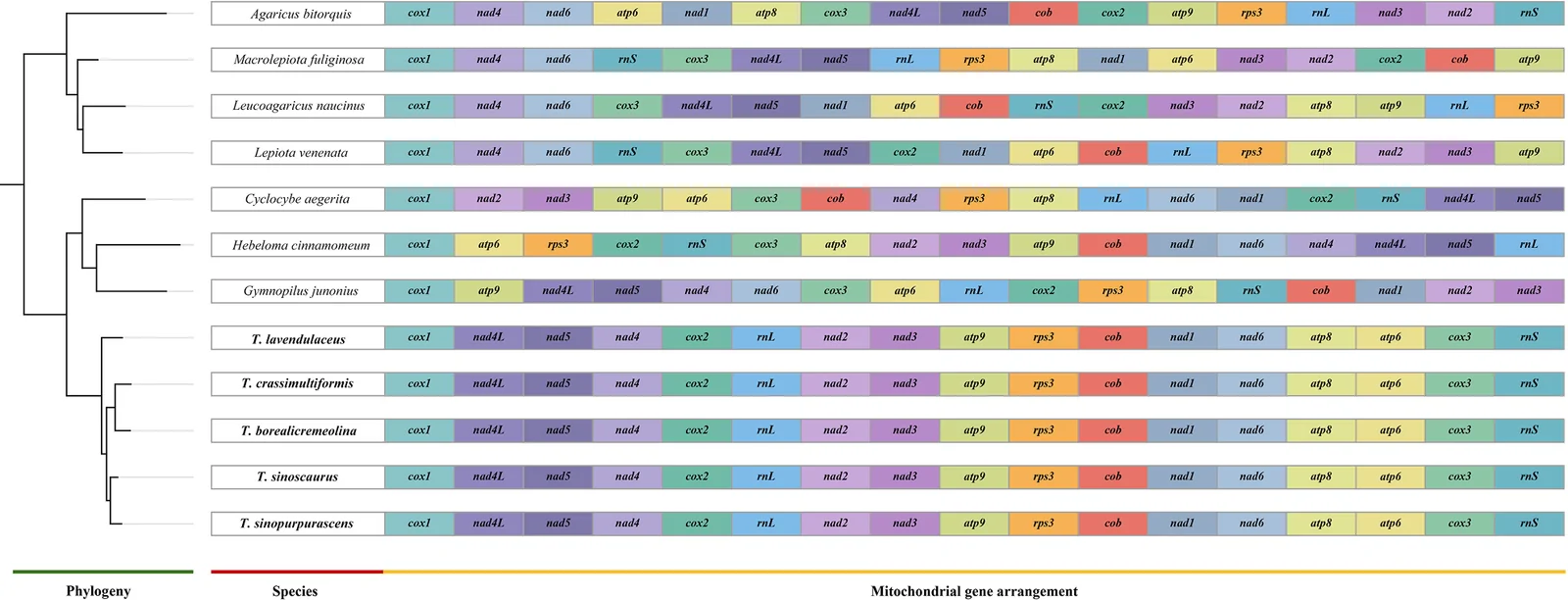
Mitochondrial gene arrangements in 12 related species. The arrangement of 15 core PCGs and two rRNA genes is compared among species. All mitogenomes are shown with *cox1* as the starting gene. The phylogenetic relationships are shown on the left, and the mitochondrial gene arrangements on the right. Different colours represent different genes.

We compared the arrangement of the 15 core PCGs and two rRNA genes across the mitogenomes of 12 related species (Fig. 5). The results revealed marked differences in mitochondrial gene order among genera, mainly reflected by changes in the relative positions of PCGs and rRNA genes. This indicates that mitochondrial gene rearrangements may have occurred to different extents across these genera. In contrast, the five *Thaxterogaster* species analysed in this study share identical gene arrangements, suggesting that the arrangement of core PCGs is well conserved within this genus.

**Figure 6.**
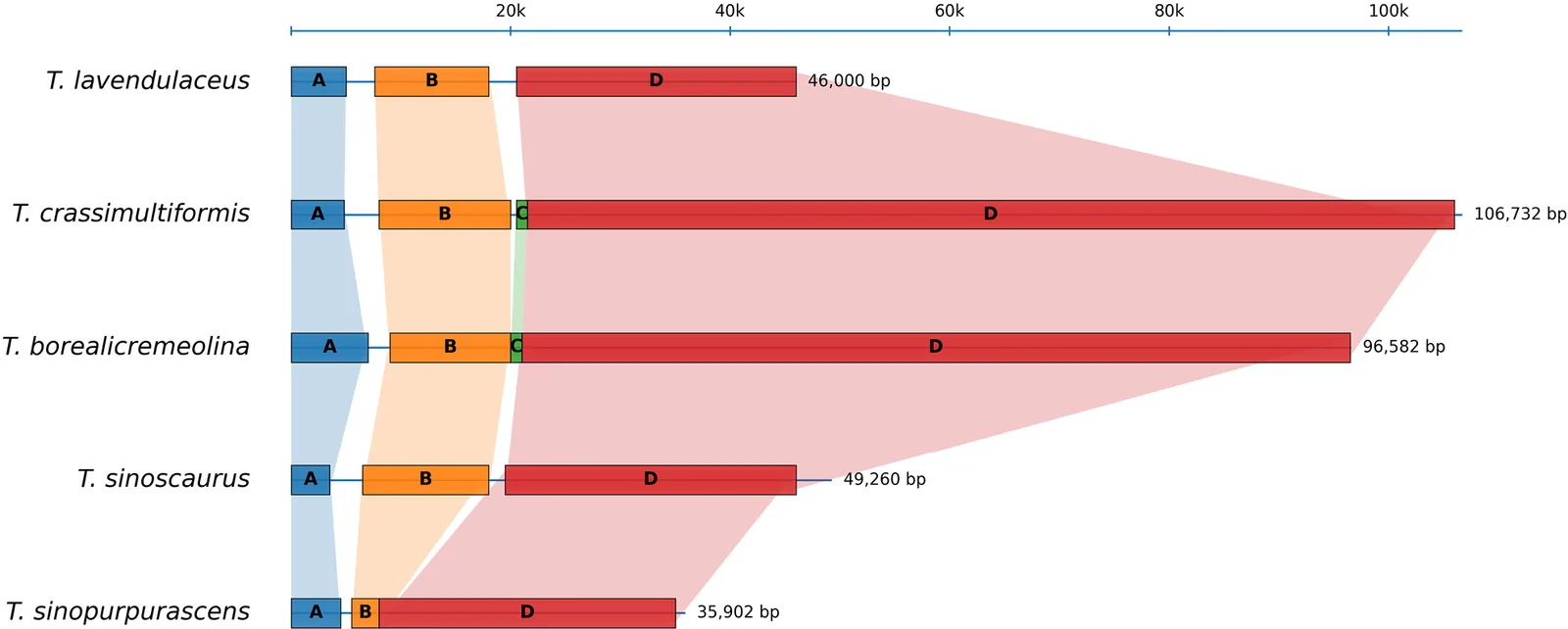
Mauve synteny analysis of the five *Thaxterogaster* mitogenomes. Four major locally homologous blocks (A–D) were identified. Rectangular blocks of the same colour indicate homologous regions shared among the mitogenomes.

Synteny analysis was performed on the complete mitogenomes of the five species (Fig. 6), revealing four major locally homologous syntenic regions (A–D). Regions A, B, and D were detected in all five species, whilst region C was exclusive to *T. crassimultiformis* and *T. borealicremeolina*. Their coordinates, genomic proportions, and annotated gene contents are provided in Suppl. material 1: table S11. Region D represented the largest syntenic block, yet its total length varied markedly among species. In detail, region D was extended in *T. crassimultiformis* and *T. borealicremeolina*, whereas shorter versions were present in *T. lavendulaceus*, *T. sinoscaurus* and *T. sinopurpurascens*. Across the 10 pairwise comparisons, aligned lengths within the shared locally collinear blocks (LCBs) ranged from 16,700 to 43,479 bp, corresponding to 17.27–64.02% of the respective mitogenomes, with nucleotide identities of 83.43–93.80% (Suppl. material 1: table S12). The greatest aligned length occurred between *T. crassimultiformis* and *T. borealicremeolina*, the only species pair sharing all four LCBs. These differences mainly reflected variation in block length, particularly block D, rather than extensive changes in block presence.

In addition, multiple synteny analysis of the complete mitogenomes of 12 related species, implemented via BLASTN and MCScanX, revealed differences in local syntenic patterns among these species (Suppl. material 2: fig. S4). Overall, although the five *Thaxterogaster* species shared an identical core gene order, differences in the lengths of local homologous regions and in syntenic patterns were still observed at the whole-mitogenome level.

### Comparative analysis of mitogenomes from related species

Comparative analysis of the 12 related mitogenomes revealed marked differences in genome size, ranging from 35,902 to 161,145 bp, with an average of 82,624 bp (Suppl. material 1: table S1). Among the five *Thaxterogaster* species examined, genome sizes ranged from 35,902 to 106,732 bp, with *T. crassimultiformis* and *T. borealicremeolina* representing the larger ones. GC content varied from 24.35% to 36.34% across the 12 mitogenomes, averaging 28.81%.

Among the 12 mitogenomes, five showed positive AT-skew values, and all showed positive GC-skew values. All species possessed the full set of 15 core PCGs, whilst the numbers of rRNA and tRNA genes ranged from 2 to 3 and from 24 to 36, respectively. Intron content was highly variable, ranging from 0 to 47 across species. Notably, *T. crassimultiformis* and *T. borealicremeolina* contained 17 and 16 introns, respectively, whereas no introns were found in *T. lavendulaceus*, *T. sinoscaurus*, or *T. sinopurpurascens*.

### Phylogenetic analysis based on mitochondrial core genes

**Figure 7.**
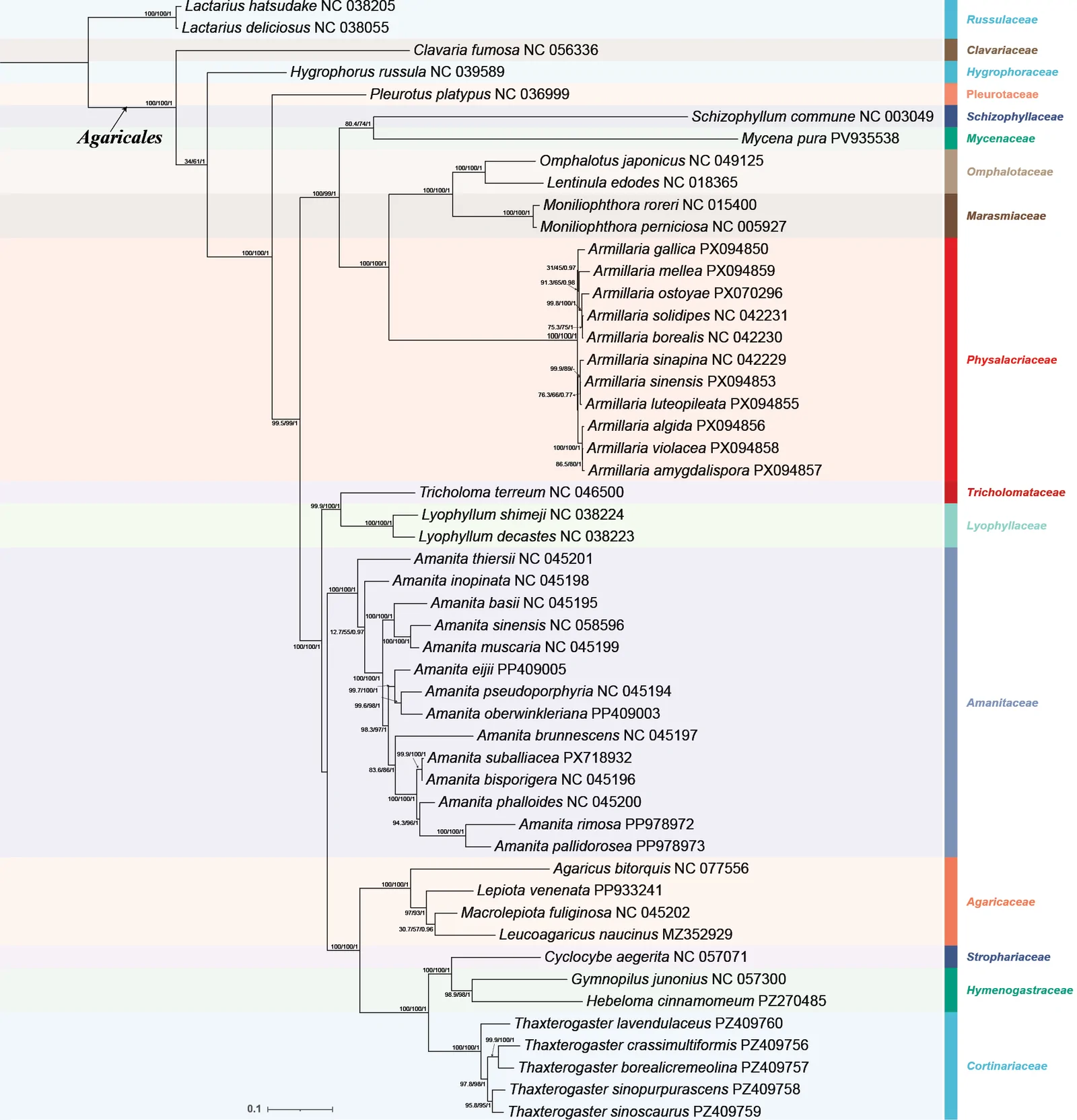
Mitogenomic phylogeny of *Agaricales* with two *Russulales* species as the outgroups. The tree was reconstructed based on the concatenated amino acid sequences of 15 core PCGs. Numbers beside branches indicate Shimodaira–Hasegawa approximate likelihood-ratio test (SH-aLRT), ultrafast bootstrap (UFBoot), and Bayesian posterior probability (BPP) support values, respectively. GenBank accession numbers are shown after species names.

Phylogenetic analysis was performed on 51 species based on the concatenated amino acid sequences of 15 core PCGs. Information on the species analysed, along with NCBI accession numbers and mitogenome features, is provided in Suppl. material 1: table S1. The topologies generated by ML and BI were largely congruent, and the ML tree was selected as the representative one (Fig. 7). The *Agaricales* species formed a well-resolved clade clearly separated from the *Russulales* outgroups, and most family-level lineages were recovered as distinct monophyletic clades.

In the resulting phylogeny, the five *Thaxterogaster* species clustered into a well-supported distinct clade, which was positioned adjacent to related lineages of *Hymenogastraceae* and *Strophariaceae*. Within the *Thaxterogaster* clade, *T. crassimultiformis* and *T. borealicremeolina* formed a fully supported sister pair, and *T. sinoscaurus* and *T. sinopurpurascens* constituted another sister subclade, with *T. lavendulaceus* occupying the basal position.

## Discussion

### Evolutionary features of *Thaxterogaster* mitogenomes

In this study, the complete mitogenomes of five *Thaxterogaster* species were sequenced, assembled, annotated and comparatively analysed. The mitogenome sizes varied considerably among these species (35,902–106,732 bp). Fungal mitochondrial genome size variation is mainly associated with changes in dynamic non-core regions, such as introns, intergenic regions and repeat sequences (Aguileta et al. 2014; Kulik et al. 2021; Kouvelis et al. 2023).

Despite substantial size variation, all five species retained the same set of 15 core PCGs, consistent with the general feature that most fungal mitogenomes maintain a relatively conserved core PCG complement (Li et al. 2020; Kulik et al. 2021). This indicates that size variation in *Thaxterogaster* mitogenomes is not mainly driven by gain or loss of core PCGs, but rather by variation in regions outside the core genes. Additionally, all five species contained two rRNA genes, and only minor variation was observed in tRNA composition. This suggests that the RNA gene composition is generally stable in *Thaxterogaster* mitogenomes, despite occasional changes in gene copy number. Similar patterns have been reported in other fungal mitogenome studies (Kulik et al. 2021; Kouvelis et al. 2023).

The predicted RNA editing patterns suggest that mitochondrial transcripts may undergo gene- and lineage-specific modification in *Thaxterogaster*. However, because these sites were predicted computationally without transcriptomic validation, their biological significance remains to be experimentally confirmed.

Overall, *Thaxterogaster* mitogenomes are characterised by a conserved core functional gene complement accompanied by variation in local gene copy number and non-core regions, which is broadly consistent with the evolutionary patterns reported for fungal mitogenomes (Li et al. 2020; Kulik et al. 2021; Kouvelis et al. 2023). However, given the currently limited sampling within the genus, the specific mechanisms underlying mitogenome size variation and non-core region variation require further validation using data from a broader range of species.

### Effects of intron dynamics on mitogenome size variation

Introns are important sources of structural and size variation in fungal mitogenomes, with mobile introns and associated accessory sequences, such as homing endonuclease genes, playing a key role in this process (Joardar et al. 2012; Megarioti and Kouvelis 2020; Ahrendt et al. 2026). This indicates that introns are unevenly distributed among mitochondrial genes and are strongly enriched in *cox1*. Similar patterns have also been observed in other fungal mitogenome studies (Férandon et al. 2010; Li et al. 2022).

Pearson correlation analysis revealed a significant positive correlation between total intron number and mitogenome size (R = 0.8465, P = 0.0005), consistent with previous findings on the role of intron dynamics in fungal genome size variation (Li et al. 2020; Song et al. 2024). Among the five *Thaxterogaster* species, *T. crassimultiformis* and *T. borealicremeolina* contained 17 and 16 introns, respectively, and their mitogenomes were markedly larger than those of the other three species. Further Pcl classification of *cox1* introns showed that *T. crassimultiformis* and *T. borealicremeolina* shared several insertion sites, while each also harbored species-specific Pcl types. This indicates that their *cox1* introns may include both commonly retained insertion sites and lineage-specific changes.

Previous studies have shown that *cox1* introns exhibit high insertion-site diversity in fungal mitogenomes, and that Pcl classification is used to compare *cox1* intron insertion positions among species (Férandon et al. 2010). It should be noted, however, that the present study focused primarily on intron number and *cox1* insertion positions, without comparing intron length, intron type, homing endonuclease composition, or sequence similarity. Therefore, the specific origins of these introns and their lineage-specific changes remain to be further investigated.

### Structural variation and mitogenome diversity

Repeat sequences are important variable components of fungal mitogenomes and are often associated with changes in mitogenome size, recombination, and local structural variation (Burger et al. 2003). In this study, a total of 192 SSRs, 94 tandem repeats, and 184 pairs of interspersed repeats were identified across the five *Thaxterogaster* species. The numbers of different repeat types varied markedly among species, indicating differential repeat accumulation within the mitogenomes of this genus. This result is consistent with previous fungal mitogenome studies showing that repeat sequences are involved in genome structural variation (Sandor et al. 2018; Song et al. 2024).

Changes in mitochondrial gene order can reflect genome rearrangement processes, and closely related fungal species often exhibit a high degree of conservation in core gene order (Sankoff et al. 1992; Song et al. 2024). Our results revealed marked differences in core gene order among genera, whereas core gene order was conserved among *Thaxterogaster* species. Further synteny analysis showed that, despite relatively conserved core gene order, the composition and length of local homologous regions still differed among the five *Thaxterogaster* species, indicating that the structural diversity in this genus may result from local fragment expansion, contraction, or retention.

### Phylogenetic applicability of mitochondrial core PCGs

Multigene and nuclear genomic data are widely used in fungal phylogenetic studies. Matheny et al. (2006) and He et al. (2019) employed multigene datasets to reconstruct phylogenetic frameworks for major lineages of *Agaricales* and *Basidiomycota*, respectively, while Wang et al. (2024) provided a framework for the major lineages of *Agaricales* based on single-copy orthologous genes from nuclear genomes. Compared with these multigene and nuclear genomic phylogenies, the tree reconstructed in this study using 15 mitochondrial core PCGs showed a high degree of congruence in its overall topology, with no obvious conflicts at the family or genus level. This indicates that, despite their distinct genetic origin, mitochondrial core PCGs can still recover the main phylogenetic relationships of the *Agaricales* taxa examined and provide effective molecular evidence for phylogenetic inference.

The five *Thaxterogaster* species analysed in this study formed a stable monophyletic clade, with interspecific relationships largely congruent with the multigene results of Wang et al. (2025). Notably, the mitogenome-based tree yielded higher nodal support values, further demonstrating the utility of mitochondrial core PCGs in resolving phylogenetic relationships within *Thaxterogaster* and closely related lineages. However, because mitochondrial genes are inherited as a single linkage group and may also be affected by compositional heterogeneity, the high support values should not be interpreted as evidence from multiple independent loci (Moore 1995; Liu et al. 2014). Nevertheless, the limited taxonomic sampling in this study cannot fully represent the mitogenomic diversity of the genus, and the inferred relationships warrant further validation using broader species sampling and combined nuclear-mitogenome datasets. In future studies, more mitochondrial genome data of *Thaxterogaster* allied genera will be incorporated into phylogenetic analyses to improve the resolution of intergeneric relationships within the *Cortinariaceae*.

## Conclusions

In this study, we sequenced and annotated the complete mitogenomes of five *Thaxterogaster* species and systematically characterised their basic features, including genome size, nucleotide composition, and gene content. The core functional gene composition was relatively conserved across the five species, with each mitogenome containing 15 core PCGs, two rRNA genes, and 25–26 tRNA genes. Comparative analyses revealed that these species shared identical core PCG complements and core gene orders, but differed markedly in genome size, intron load, repeat sequence composition, and local syntenic regions. Phylogenetic reconstruction based on the 15 core PCGs demonstrated that mitochondrial core protein-coding genes are phylogenetically informative for resolving relationships within *Thaxterogaster* and its close relatives. Overall, this study enriches the currently limited mitogenomic resources for *Thaxterogaster* and provides a valuable reference for future studies into mitogenome evolution and phylogeny in *Cortinariaceae* and related groups.

## Additional information

### Artificial Intelligence (AI) use

The authors accept full responsibility for the content of the manuscript, including the disclosure of any use of AI.

No AI tools were used in the preparation of this manuscript.

### Author ORCIDs

L.-Y. Ou https://orcid.org/0009-0005-9943-8727

M.-L. Xie https://orcid.org/0000-0002-7798-7048

Y. Li https://orcid.org/0000-0002-3482-5487

C.-T. Li https://orcid.org/0000-0001-6498-8642

### Data availability

The complete mitogenomes and nrITS sequences of the five *Thaxterogaster* species have been deposited in GenBank. The accession numbers are provided below: *T. borealicremeolina* (mitogenome PZ409757, nrITS PZ575560), *T. crassimultiformis* (mitogenome PZ409756, *nrITS*PZ571176), *T. lavendulaceus* (mitogenome PZ409760, *nrITS*PZ571177), *T. sinopurpurascens* (mitogenome PZ409758, *nrITS*PZ575561), and *T. sinoscaurus* (mitogenome PZ409759, nrITS PQ423063). The phylogenetic alignment has been submitted to TreeBASE under Study S32755. All data generated or analysed during this study are available in the main manuscript and Suppl. materials 1, 2.

## Supplementary materials

Supplementary material 1 Supplementary tables Data type xlsx Explanation note **table S1**. Species information, GenBank accession numbers and mitochondrial genome features used for the phylogenetic analysis in this study. **table S2**. Gene information encoded by the mitochondrial genomes of five *Thaxterogaster* species. **table S3**. RSCU values of codons in the mitochondrial genomes of five *Thaxterogaster* species. **table S4**. SSR loci detected in the mitochondrial genomes of five *Thaxterogaster* species. **table S5**. Data analysis of SSRs in the mitochondrial genomes of five *Thaxterogaster* species. **table S6**. Tandem repeats in the mitochondrial genomes of five *Thaxterogaster* species. **table S7**. Interspersed repeats in the mitochondrial genomes of five *Thaxterogaster* species. **table S8**. Potential RNA editing sites in PCGs of the mitochondrial genomes of five *Thaxterogaster* species. **table S9**. Amino acid changes in the potential RNA editing sites of PCGs in the mitochondrial genomes of five *Thaxterogaster* species. **table S10**. Statistical evaluation of predicted mitochondrial RNA editing patterns in the five species examined. **table S11**. Coordinates, lengths, genomic proportions, orientations, and annotated contents of the four locally collinear blocks in five *Thaxterogaster* mitochondrial genomes. **table S12**. Quantitative pairwise homology among the five *Thaxterogaster* mitochondrial genomes based on the Mauve alignment. https://binary.pensoft.net/file/1779739This dataset is made available under the Open Database License (http://opendatacommons.org/licenses/odbl/1.0/). The Open Database License (ODbL) is a license agreement intended to allow users to freely share, modify, and use this Dataset while maintaining this same freedom for others, provided that the original source and author(s) are credited. Li-Ye Ou, Meng-Le Xie, Yi Li, Chang-Tian Li

Supplementary material 2 Supplementary figures Data type docx Explanation note **fig. S1**. Putative secondary structures of tRNA genes identified in the mitochondrial genomes of five *Thaxterogaster* species. **fig. S2**. Length, GC content, and nucleotide skews of mitochondrial core PCGs in five *Thaxterogaster* species. **fig. S3**. Distribution of repeats in the mitochondrial genomes of five *Thaxterogaster* species. **fig. S4**. Collinearity analysis among the 12 mitochondrial genomes. **fig. S5**. Characteristics of predicted RNA editing sites in mitochondrial PCGs of five *Thaxterogaster* species. https://binary.pensoft.net/file/1779740This dataset is made available under the Open Database License (http://opendatacommons.org/licenses/odbl/1.0/). The Open Database License (ODbL) is a license agreement intended to allow users to freely share, modify, and use this Dataset while maintaining this same freedom for others, provided that the original source and author(s) are credited. Li-Ye Ou, Meng-Le Xie, Yi Li, Chang-Tian Li
