# Supplementary material for: Assembly and comparative analysis of the complete mitochondrial genomes of five Thaxterogaster species (Cortinariaceae, Basidiomycota): Insights into mitochondrial genome evolution and intrageneric phylogenetic relationships
Source: MycoKeys. 2026 Sep 18;140:217–39. doi: 10.3897/mycokeys.140.205520 (PMC13615393; doi:10.3897/mycokeys.140.205520)
Supplement: Supplementary file 1 [file mycokeys-140-217-s002.docx]

**Figure S1**. Putative secondary structures of tRNA genes identified in the mitochondrial genomes of five *Thaxterogaster* species. The tRNAs in green font represent tRNAs shared by the five *Thaxterogaster* species, while the tRNA in blue font represents a tRNA found only in *T. crassimultiformis*. Residues conserved across the five mitochondrial genomes are shown in green, while variable sites are shown in red. All genes are shown in their orders of occurrence in the mitochondrial genome of *T. crassimultiformis* starting from trnS-1. Suffixes -1, -2 and -3 indicate duplicated tRNA copies.

**Figure S2.** Length, GC content, and nucleotide skews of mitochondrial core PCGs in five *Thaxterogaster* species. A, lengths of the 15 core PCGs; B, GC content of each core PCG; C, AT-skew of each core PCG; D, GC-skew of each core PCG.

**Figure S3.** Distribution of repeats in the mitochondrial genomes of five *Thaxterogaster* species. The outermost circle represents SSRs, followed by tandem repeats, while the lines in the innermost circle represent interspersed repeats. Numbers around each circle indicate genome coordinates. Panels A–E correspond to *T. borealicremeolina*, *T. crassimultiformis*, *T. lavendulaceus*, *T. sinopurpurascens*, and *T. sinoscaurus*, respectively. For visualization, short SSR and tandem repeat regions were displayed with a minimum track width while retaining their relative genomic positions.

**Figure S4.** Collinearity analysis among the 12 mitochondrial genomes. Colored bars represent complete mitochondrial genomes, while ribbons indicate collinear homologous regions identified between adjacent species.

**Figure S5.** Characteristics of predicted RNA editing sites in mitochondrial PCGs of five *Thaxterogaster* species. A. Distribution of predicted C-to-U RNA editing sites among mitochondrial PCGs. The x-axis represents mitochondrial protein-coding genes, and the y-axis represents the number of predicted RNA editing sites. B. Statistics of amino acid conversion types potentially caused by RNA editing events. The x-axis represents amino acid changes, and the y-axis represents the number of occurrences for each change type.
